# A Retrospective Hospital-Based Study of HMGCR Expression in HER2 IHC 2+ and 3+ Breast Cancer

**DOI:** 10.31557/APJCP.2021.22.7.2043

**Published:** 2021-07

**Authors:** Malisalaora Mohamed, Siti Norasikin Mohd Nafi, Hasnan Jaafar, Noorasmaliza Md Paiman

**Affiliations:** 1 *Department of Pathology, School of Medical Sciences, Health Campus, Universiti Sains Malaysia, Kubang Kerian, Kelantan, Malaysia. *; 2 *Hospital Universiti Sains Malaysia, Health Campus, Universiti Sains Malaysia, Kubang Kerian, Kelantan, Malaysia. *; 3 *Department of Pathology, Hospital Sultanah Bahiyah, Bandar Alor Setar, Alor Setar, Kedah, Malaysia. *

**Keywords:** HER2 IHC 2+, HER2 IHC 3+, HMGCR

## Abstract

**Objective:**

The role of HMG-CoA reductase (HMGCR) in relation to prognostic and treatment predictive information of HER2 positive breast cancer has been newly explored. In this study, we aimed to determine the expression of HMGCR in HER2 immunohistochemistry (IHC) scores of 2+ and 3+ breast cancer and to correlate with the patients’ outcomes.

**Methodology:**

Using a cross-sectional design, invasive breast carcinoma of no special type (NST) and HER2 IHC scores of 2+ and 3+ cases were selected over a 50-month period in Hospital Sultanah Bahiyah (HSB), Alor Setar. IHC staining for HMGCR was performed on paraffin-embedded tissues at the Pathology Laboratory, Hospital Universiti Sains Malaysia (HUSM), Kubang Kerian using the standard staining procedure. The results were correlated with the patient’s demographic and clinicopathological data.

**Results:**

A total of 59 cases of HER2 IHC 2+ and 3+ invasive breast carcinoma were identified. The cases were predominant in young Malay women with tumours smaller than 50mm, higher grade and positive for lymphovascular invasion, axillary lymph nodes involvement and ER/PR expressions. HMGCR was positively expressed in HER2 IHC 2+ and 3+ breast cancer cases, which the staining intensities varied from weak, moderate to strong. Majority of the cases were scored 1+ for HMGCR expression. A low-positive HMGCR was more likely to be associated with less favourable outcomes of patients with HER2 IHC 2+ and 3+. However, the associations were statistically not significant.

**Conclusion:**

A study in a larger cohort of tumour samples is needed to further validate HMGCR expression as a potential prognostic biomarker for HER2 positive breast cancer. It is also suggested that all the HER2 IHC 2+ and 3+ cases need to be gene amplified using FISH analysis.

## Introduction

The Malaysia National Cancer Registry Report stated an increase in female cancer cases in Malaysia from 2012-2016 (Registry, 2019). From the report, a total of 3,815 cases of female cancer were diagnosed for a five-year period in Kedah, a northern state of Malaysia. Among all female cancer subtypes, breast cancer has been reported the most common cancer among Malaysian population (Observatory, 2020). Approximately 30% of Malaysian female breast cancer patients are HER2-positive, which categorises an aggressive subtype (Tan et al., 2009).

The interpretation of HER2 positive breast cancer is made through IHC staining for the HER2 protein expression and fluorescence in situ hybridization (FISH) for amplification of HER2 gene (Gutierrez and Schiff, 2011). Research on the pathogenesis of HER2 positive breast cancer has embarked treatment options to combat this poor prognosis for many women. The utilization of trastuzumab, a monoclonal anti HER2 is the foundation of systemic treatment of HER2 positive breast cancer (Gajria and Chandarlapaty, 2011). Regardless of its noteworthy effect on HER2 positive breast cancer, resistance to trastuzumab remains a challenge, requesting further research to be done.

Previous study suggests high cholesterol as a risk factor for breast cancer (Murai, 2015). The cholesterol biosynthesis regulator, HMGCR catalyses the conversion of HMG-Co A to mevalonic acid, which is a crucial step in the biosynthesis of cholesterol (Zeki et al., 2017). To date, HMGCR remains to expand beyond its direct role in cholesterol synthesis following the discovery that HMGCR promotes breast cancer development and progression (Zhou and Liao, 2009; Murai, 2015). Inhibiting HMGCR via simvastatin treatment effects on cholesterol reduction and represents a common treatment for cardiovascular disease (Zeki et al., 2017). The treatment was seen to induce anti-carcinogenic effects by increasing apoptosis and inhibiting inflammation, proliferation, migration and angiogenesis (Gustbee et al., 2015).

A current study have evaluated the role of HMGCR in relation to prognostic and treatment predictive information of HER2 positive breast cancer (Sethunath et al., 2019). In this study, we aimed to determine the expression of HMGCR in HER2 IHC scores of 2+ and 3+ breast cancer and to correlate the expression with the patients’ outcomes.

## Materials and Methods

*Patient data*

This was a cross sectional study carried out at HSB for a period of 50 months. This study was approved by the Human Research Ethics Committee of USM (USM/JEPeM/17050276) and the Medical Research & Ethics Committee (NMRR-17-1303-35997). Selection of cases was based on the confirmed diagnosis of invasive breast carcinoma of NST with HER2 IHC scores of 2+ (HER2 equivocal) and 3+ (HER2 overexpression). The demographic data and tumour characteristics from the pathology reports were acquired from the archived Electronic Hospital Information System (e-HIS) of HSB. The clinicopathological data for the assessment include age, race, tumour size, tumour grade (according to modified Bloom and Richardson), the lymph node involvement status as well as extranodal involvement, lymphovascular invasion and hormonal status (estrogen receptor, ER and progesterone receptor, PR). The tumour grading characteristics evaluation using modified Bloom and Richardson criteria were the degree of tubule formation, nuclear pleomorphism and mitotic counts. Paraffin embedded tissue blocks of breast cancers were collected from the Department of Pathology, HSB. To protect confidentiality of patients, all tissue blocks and clinicopathological data were assigned with pathological numbers.

*IHC staining of HMGCR*

The IHC staining of HMGCR was performed at the Pathology Laboratory, HUSM using the standard staining procedure. The paraffin blocks were sectioned at 4 µm of thickness and subjected to IHC staining using the REAL™ EnVision™ Detection System Peroxidase-DAB kit (DAKO, Cat# K5007). To eliminate endogenous peroxidase activity, tissue sections were deparaffinized, rehydrated and incubated with 3% H_2_O_2_ in methanol for 15 min at room temperature. The antigen was retrieved by placing the slides in target retrieval solution at 95°C for 20 min. The slides were then incubated with HMGCR antibody (Thermo Fisher Scientific, Cat# PA5-37367) at 4°C overnight. After incubation with secondary antibody at room temperature for 30 min, the slides were incubated with HRP-peroxidase complex at room temperature for 30 min. Reaction products were visualised using 3,3-diamiobenzidine (DAB) (DAKO, Cat# K5007) and haematoxylin solution (Merck, Cat# 105174) was used for counterstaining. The gallbladder mucosa was used as the positive tissue control. Omission of the primary antibody was used as an additional negative control.

*HMGCR scoring*

Results were interpreted by two pathologists who were blinded to the specific diagnosis and prognosis for each case (Gustbee et al., 2015). For HMGCR staining, staining intensities were scored as no staining (0), weak staining (1+), moderate staining (2+), or strong staining (3+). The percentage of staining areas was classified as 0, 0%; 1, 1–10%; 2, 11–50%; 3, 51–100%. Following the IHC scoring, the intensity and percentage scores were multiplied to give composite scores of 0–9 for each specimen (Kaemmerer et al., 2012). Composite scores of 0–3 were defined as having low HMGCR protein expression and scores of 4–9 were considered to be high expression of HMGCR. 

*Statistical analysis*

Statistical calculations were performed using the SPSS software package version 24.0. The IHC profiles were compared to the clinicopathological parameters using the Pearson Chi-Square and Fisher’s exact tests. A two-tailed test was applied to test the statistical significance level at or below 5% (0.05).

## Results

*Characteristics of HER2 IHC scores of 2+ and 3+ breast cancer patients*

A total of 59 breast cancer cases with IHC scores of 2+ and 3+ were enlisted into this study. Majority of patients were in the HER2 3+ group (38/59, 64.4%) while the others were in the HER2 2+ group (21/59, 35.6%, Table 1). Clinicopathological characteristics of the patients are shown in Table 2. Overall, HER2 IHC scores of 2+ and 3+ breast cancer patients were predominant in Malay (54/59, 91.5%), of the age group of less than 55 years (31/59, 52.5%), and of tumour size between 20 mm to 49 mm (34/59, 57.6%). There were 38 (64.4 %) cases of tumour grade 3, which made up the most prevalent tumour grades of HER2 IHC scores of 2+ and 3+ breast cancer. 

Of these 59 cases, 28 (47.5%) showed lymphovascular invasion. In addition, extranodal extension was found in 42.4% (25/59) of patients with HER2 IHC scores of 2+ and 3+. In the present study, axillary lymph nodes were positive in 38/59 (64.4%) of breast cancer cases with IHC scores of 2+ and 3+, with majority regional lymph nodes subtypes of pN1a and pN2a (Table 2). Additionally, positive expressions of ER and PR were noted in 62.7% (37/59) and 44.1% (26/59%) of cases, respectively (Table 2).

*Expression of HMGCR in HER2 IHC scores of 2+ and 3+ breast cancer*

**Figure 1:**
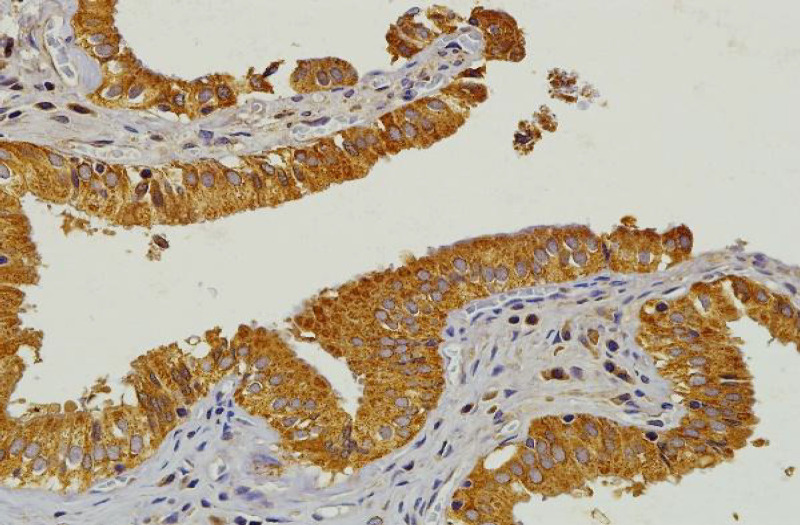
IHC Staining for HMGCR in Normal Gallbladder Mucosa. Strong positive expression of HMGCR in gallbladder mucosa (positive control) manifested as brown staining in the cytoplasm. Original magnification, 200x

**Figure 2:**
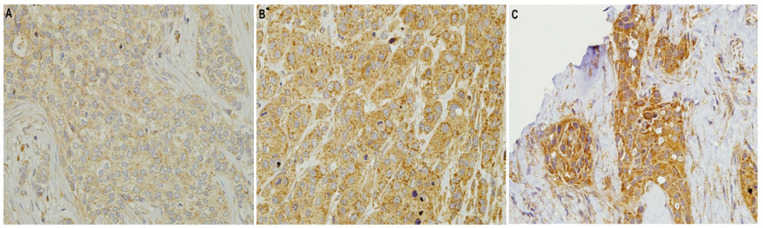
HMGCR Expression in HER2 IHC Scores of 2+ and 3+ Breast Cancer. Representative patterns of HMGCR cytoplasmic staining intensity: (A) 1+, weak expression (B) 2+, moderate expression (C) 3+, strong expression. Original magnification, 200x

Positive control of gallbladder tissue presented clear and strong cytoplasmic staining for HMGCR at dilutions of 1:100 for 24-hour incubation (Figure 1). HMGCR staining intensities showed weak, moderate to strong cytoplasmic positivity in HER2 IHC scores of 2+ and 3+ breast cancer cases (Figure 2). HMGCR staining was weakly positive (1+) in the majority of HER2 IHC scores of 2+ and 3+ breast cancer cases (33/59, 55.9%, Table 3). 16 out 59 cases (27.1%) and 10 out 59 cases (16.9%) of HER2 IHC scores of 2+ and 3+ breast cancer cases were scored with moderate 2+ and strong 3+ HMGCR, respectively. 

*Relation between HMGCR expression and clinicopathological features of HER2 IHC scores of 2+ and 3+ breast cancer patients*

Table 4 demonstrates the analysis of clinicopathological features associated with HMGCR expression from 59 patients with HER2 IHC scores of 2+ and 3+ breast cancer. It was noted that low-positive HMGCR group was more likely to be of larger tumour size, higher grade, lymphovascular invasion, extranodal extension and axillary lymph node involvement, and ER negative. However, there was no significant association between HMGCR expression with all demographic and clinicopathological characteristics of the breast cancer patients (Table 4).

**Table 1:** HER2 IHC Scores of 2+ and 3+ Cases

	Breast cancer patients
HER2 IHC score	n (%)
2+	21 (35.6)
3+	38 (64.4)

**Table 2:** Demographic and Clinicopathological Characteristics of HER2 IHC scores of 2+ and 3+ Patients (n=59)

	HER2 IHC scores of 2+ and 3+ cases
Variables	n (%)
Race	
Malay	54 (91.5)
Chinese	3 (5.1)
Indian	2 (3.4)
Age	
34 to 54 years	31 (52.5)
55 years and above	28 (47.5)
Invasive Tumour Size (mm)	
Less than 20mm	9 (15.3)
20 to 49mm	34 (57.6)
50mm and above	16 (27.1)
Tumour Grade	
1	3 (5.1)
2	18 (30.5)
3	38 (64.4)
Lymphovascular Invasion	
No	31 (52.5)
Yes	28 (47.5)
Extranodal Extension	
No	34 (57.6)
Yes	25 (42.4)
Axillary Lymph Node Involvement	
No	21 (35.6)
Yes (number of positive nodes out of the total)	38 (64.4)
pN1a (1 to 3 positive nodes)	16 (42.1)
pN2a (4 to 9 positive nodes)	15 (39.5)
pN3a (10 positive nodes and above)	7 (18.4)
Estrogen	
Negative	22 (37.3)
Positive	37 (62.7)
Progesterone	
Negative	33 (55.9)
Positive	26 (44.1)

**Table 3:** Evaluation of IHC Staining of HMGCR in HER2 IHC Scores of 2+ and 3+ Breast Cancer

HMGCR score	HER2 IHC scores of 2+ and 3+ cases (n=59) n (%)
0	0 (0.0)
1+	33 (55.9)
2+	16 (27.1)
3+	10 (16.9)

**Table 4:** Analysis of Clinical Factors Associated with HMGCR Expression from 59 Patients with HER2 IHC Scores of 2+ and 3+ Breast Cancer

	Low	High	
Variables	n (%)	n (%)	p value
Race			
Malay	30 (55.6)	24 (44.4)	0.495 ^b^
Chinese	1 (33.3)	2 (66.7)	
Indian	2 (100.0)	0 (0.0)	
Age			
34 to 54 years	18 (58.1)	13 (41.9)	0.728 ^a^
55 years and above	15 (53.6)	13 (46.4)	
Invasive Tumour Size (mm)			
Less than 20mm	5 (55.6)	4 (44.4)	0.460 ^a^
20 to 49mm	17 (50.0)	17 (50.0)	
50mm and above	11 (68.8)	5 (31.3)	
Tumour Grade			
1	1 (33.3)	2 (66.7)	0.668 ^b^
2	11 (61.1)	7 (38.9)	
3	21 (55.3)	17 (44.7)	
Lymphovascular Invasion			
No	18 (58.1)	13 (41.9)	0.728 ^a^
Yes	15 (53.6)	13 (46.4)	
Extranodal Extension			
No	18 (52.9)	16 (47.1)	0.589 ^a^
Yes	15 (60.0)	10 (40.0)	
Axillary Lymph Node Involvement			
No	13 (61.9)	8 (38.1)	0.492^ a^
Yes	20 (52.6)	18 (47.4)	
ER			
Negative	13 (59.1)	9 (40.9)	0.706 ^a^
Positive	20 (54.1)	17 (45.9)	
PR			
Negative	17 (51.5)	16 (48.5)	0.441 ^a^
Positive	16 (61.5)	10 (38.5)	
HER2			
2+	6 (28.6)	15 (71.4)	0.075 ^a^
3+	20 (52.6)	18 (47.4)	

^a^, Pearson Chi-Square test; ^tb^, Fisher’s exact test

## Discussion

The upregulation of HMGCR, a cholesterol synthesis regulator has been formerly shown to be modulated by tyrosine kinase activity of HER2 (Asslan et al., 1999). The study suggested the association between HMGCR expression and HER2. In addition, HMGCR inhibition via simvastatin treatment induced HER2 repression and cell death in HER2 positive breast cancer cells (Zhao et al., 2012). Inhibiting HMGCR via statin was shown to give a preventive effect and the reversible mechanism on tumour progression (Borgquist et al., 2008).

We carried out a retrospective hospital-based study to determine the expression of HMGCR in HER2 IHC scores of 2+ and 3+. The IHC is the initial test for HER2 protein expression with results are normally divided into four scale scores (0, 1+, 2+ and 3+), depending on the staining intensity and the percentage of positive tumor cells. HER2 IHC scores of 0 and 1+ have been considered as HER2 negative and those with HER2 (3+) score has been regarded as HER2 positive (Gutierrez and Schiff, 2011). However, HER2 equivocal invasive breast cancer are those with HER2 IHC 2+ score that has to be further assessed by FISH, a test that is more accurate and reliable than IHC. In our local scenario, FISH is considered as a sophisticated analysis and samples are usually sent out to an advanced diagnostic laboratory. The procedure normally takes longer time and expensive.Therefore, the tissue samples for this study were labelled as HER2 IHC 2+ and 3+ because the FISH results were not available for those HER2 IHC equivocal samples. A total of 59 cases of HER2 IHC 2+ and 3+ invasive breast carcinoma were identified, which the cases were predominant in young Malay women with tumours smaller than 50mm, higher grade and positive for lymphovascular invasion, axillary lymph nodes and ER/PR expressions. 

From a previous study, HMGCR staining was evaluated based on cytoplasmic and membranous positivity (Borgquist et al., 2008). Our study revealed that all HER2 IHC scores of 2+ and 3+ cases were observed with positive cytoplasmic HMGCR expression, which the staining intensities varied from weak, moderate to strong cytoplasmic positivity (Figure 2). Of all cases, more than 50% of HER2 IHC 2+ and 3+ cases were scored with HMGCR intensity of 1+ (Table 3).

As compared to the HMGCR membranous staining, HMGCR cytoplasmic expression was observed to be associated with more favourable parameters of breast cancer i.e. smaller tumour size, lower histological grade, low ER positivity (Borgquist et al., 2008). Additionally, moderate/strong cytoplasmic HMGCR expression was significantly associated with less axillary lymph node involvement, lower histological grade, ER and PR positivity, HER2 negativity and older patient age at the diagnosis (Gustbee et al., 2015). Our study noted that low-positive HMGCR was more likely to be associated with unfavourable outcomes of patients with HER2 IHC 2+ and 3+ (Table 4). However, we were unable to prove any significant association of the above findings. 

To highlight the limitation, it should be noted that our study involved a small sample size. In addition, all cases were selected according to HER2 status based on IHC results and were not confirmed by FISH HER2 expression. To conclude, a study with a larger cohort of tumour samples is needed to further validate HMGCR as a potential prognostic biomarker for HER2 positive breast cancer. It is also suggested that all the HER2 cases with IHC 3+ and 2+ need to be confirmed by gene amplification using FISH analysis.

## Author Contribution Statement

MM conducted the database searches and samples collection, and performed IHC experiments. SNMN planned and designed the research. HJ and NMP provided methodological support/advice. All authors read, critically reviewed and approved the final manuscript.

## Acknowledgements

### Funding

This research was supported by the Ministry of Higher Education (MHE) Fundamental Research Grant Scheme (FRGS) No. 203/PPSP/6171197. 

### Thesis

This article is a part of the thesis belong to the first author, which was approved for the Master of Pathology at the School of Medical Sciences, USM.

### Ethical approval

The ethical approvals of this research were obtained from the Human Research Ethics Committee of USM and the Medical Research & Ethics Committee of Ministry of Health (MOH) Malaysia.

### Research facilities

We thank to the management of the HUSM and the HSB for granting the permission to the authors to use patients’ medical record; space and assets belong to the hospital during the process of conducting the research.

### Technical support

We thank to all members of Pathology Lab, HUSM for providing technical assistance.

### Conflicts of interest

The authors declare that there are no competing interests associated with this article.
